# Circulating Epigenetic Biomarkers in Malignant Pleural Mesothelioma: State of the Art and critical Evaluation

**DOI:** 10.3389/fonc.2020.00445

**Published:** 2020-04-03

**Authors:** Luca Ferrari, Michele Carugno, Carolina Mensi, Angela Cecilia Pesatori

**Affiliations:** ^1^EPIGET LAB, Department of Clinical Sciences and Community Health, Università degli Studi di Milano, Milan, Italy; ^2^Epidemiology Unit, Department of Preventive Medicine, Fondazione IRCCS Ca' Granda Ospedale Maggiore Policlinico, Milan, Italy

**Keywords:** microRNA, DNA methylation, epigenetics, circulating biomarkers, mesothelioma

## Abstract

Malignant pleural mesothelioma (MPM) is a rare and aggressive cancer, which originates from the mesothelial cells of the pleura and is associated with asbestos exposure. In light of its aggressive nature, late diagnosis and dismal prognosis, there is an urgent need for identification of biomarkers in easily accessible samples (such as blood) for early diagnosis of MPM. In the last 10 years, epigenetic markers, such as DNA methylation and microRNAs (miRNAs), have gained popularity as possible early diagnostic and prognostic biomarkers in cancer research. The aim of this review is to provide a critical analysis of the current evidences on circulating epigenetic biomarkers for MPM and on their translational potential to the clinical practice for early diagnosis and for prognosis.

## Introduction

Malignant Mesothelioma is a rare cancer originating from the mesothelial cells of pleura (i.e., Malignant Pleural Mesothelioma, MPM; 80–90%), peritoneum (10–15%), and pericardium (<5%). It is characterized by a long latency period (≥30–60 years) and non-specific symptoms, and thus often implicate late diagnosis and poor survival [1].

MPM presents with heterogeneous histological features, and can be classified in three main histological subtypes, depending on cellular morphology and biological markers: epithelioid mesothelioma represents the most common form (50–70% of cases) and shows polygonal, oval, or cuboidal cells similar to carcinomas; fibrous or sarcomatoid type (10–20%) is characterized by spindle cell morphology similar to those of sarcomas; the mixed or biphasic subtype (30%) presents both the epithelioid and sarcomatoid components, in different proportions [2]. High levels of cytokeratin 5 are expressed in most mesotheliomas regardless of subtype; epithelioid mesothelioma expresses high levels of calretinin, while sarcomatoid mesothelioma does not [3]. If compared to sarcomatoid mesothelioma, the epithelioid subtype is less aggressive, highly sensitive, and more responsive to chemotherapy, thus resulting in a longer survival [4, 5].

Although MPM is considered a rare malignancy (prevalence 1–9/100,000), about 40,000 deaths have been estimated to occur each year globally for asbestos-related exposures [6, 7]. Asbestos refers to a group of naturally occurring mineral silicate fibers classified into two main families, the serpentines and the amphiboles [1]. The serpentines consist of chrysotile with characteristic short and curly fibers, also called “white asbestos,” and account for 95% of asbestos in commercial use. The amphiboles, with straight and longer fibers, include crocidolite or “blue asbestos,” amosite, tremolite, actinolite, and anthophyllite [1, 8]. The World Health Organization estimates that 125 million people annually around the world are exposed to asbestos. The International Agency for Research on Cancer confirmed that all fibrous forms of asbestos are carcinogenic to humans, causing mesothelioma, cancer of the lung, larynx, and ovary. Evidences are limited in humans for pharynx, stomach, and colorectal cancers [9].

Exposure to asbestos other than in the occupational setting affects also the families of asbestos-workers, people living close to places where asbestos is mined or processed, and the populations exposed to the fragmentation of asbestos artefacts [10–13].

The observation that mesothelioma develops in a minority of asbestos-exposed individuals [14] suggests a sort of hyper-susceptibility to asbestos carcinogenic potential, probably due to the combination of environmental exposures and genetic susceptibility [15]. Various studies have shown an association between MPM and the oncogenic simian virus 40 [SV40; [16, 17]], suggesting a transforming synergic action of asbestos and SV40 [18, 19] however the evidence supporting this association is still controversial [20, 21]. Exposure to ionizing radiations seems to play a strong role in the development of mesothelioma [22, 23]. Other environmental exposures reported as risk factors for MPM are erionite in Turkey and fluoro-edenite in Italy [24]. On the other hand, a recent breakthrough in the study of mesothelioma susceptibility has emerged. Indeed, germline mutations in different genes mainly involved in DNA damage repair confer moderate-to-high genetic risk of MPM development [25]. The BAP1-tumor predisposition syndrome is the most studied genetic condition associated with MPM development and is caused by mutations in the BRCA1-associated protein 1 (*BAP1*) gene [14, 26–32]. This new evidence should be taken into account together with environmental/occupational exposures, as it may lead to a different risk stratification.

Although the etiology of MPM is well consolidated, patients are usually diagnosed in an advanced phase, when radical surgery cannot be performed, and the current available chemotherapy is not effective, thus leading to poor survival [6, 33].

Currently, diagnosis is invasive and relies mainly on morphological analysis, with malignant growths characterized by deep stromal invasion with dense cells and complex growth patterns [2]. Although immunohistochemical markers have improved in recent years, the lack of biomarkers able to discriminate between the different subtypes continues to hamper the diagnosis of MPM [34]. Recently, high-throughput analyses have uncovered key genomic and epigenomic alterations driving MPM [35, 36].

In this context, epigenetic markers such as DNA methylation and microRNAs (miRNAs) are emerging as promising biomarkers for several cancer types, including MPM. While genetic biomarkers may differ from case to case in most cancer patients (i.e., each patient may carry a different mutation within the same gene), different subjects show variable levels of epigenetic biomarkers in a specific target depending on health/disease status [37, 38].

All the above supports the need for identification of appropriate biomarkers that can be easily evaluated in accessible tissues, in order to detect the disease at earlier stages and improve prognosis. High-throughput molecular profiling of tumors, blood, and pleural fluids is currently shedding light on novel candidate biomarkers for early diagnosis, and revealing potential new therapeutic targets for improved treatment.

The purpose of this review is to provide a critical analysis of the available evidence on circulating epigenetic biomarkers for MPM and on their effective validity for early diagnosis or prognosis.

## What Are Circulating Epigenetic Biomarkers?

The development of non-invasive methods to diagnose and monitor tumors is a major challenge in oncology. The analysis of liquid samples such as plasma, serum, urine, or cerebrospinal fluid (also known as liquid biopsy) is a suitable approach for the characterization of markers related to cancer progression, as these biological fluids are easy to collect [39–41].

An ideal biomarker for cancer detection should be easily and cheaply measurable and should allow to identify the disease at an early stage [42]. Circulating biomarkers can be released into the bloodstream by cells of different origins [e.g., Peripheral Blood Mononuclear Cells (PBMCs), and cancer cells] through various mechanisms, such as necrosis, apoptosis, or release of Extracellular vesicles (EVs) [43]. While circulating biomarkers derived from cancer cells could potentially allow the identification of a pathological condition with high specificity, those deriving from PBMCs may show higher sensitivity in pointing out the global response of the organism toward a detrimental condition.

Besides numerous protein markers largely investigated in MPM [mesothelin, osteopontin, fibulin, HMGB1 protein, etc.; [44–48]] also DNA, mRNA, and miRNA are released by cells and circulate in the blood of cancer patients. In this context, the unique properties of epigenetic biomarkers, expecially DNA methylation and miRNA expression, make them well suited as promising diagnostic and prognostic tools. DNA methylation is stable for a long time and miRNAs are particularly resistant to RNase-degradation. Moreover, aberrant peripheral epigenetic modifications have been frequently observed in early-stage cancer patients [38, 43, 49, 50]. Thus, circulating epigenetic biomarkers are promising and dynamic markers for early cancer detection, progression, and response to therapy.

## DNA Methylation

DNA methylation involves the addition of a methyl group to the fifth carbon of the cytosine base, forming a 5-methyl-cytosine [51]. DNA methylation occurring in gene promoters can directly alter gene expression, by inhibiting the access to DNA of the transcriptional machinery function. This methylation is often referred to as “gene-specific methylation” [52]. On the other hand, methylation taking place in repetitive transposable elements (e.g., Alu, LINE-1, etc.) favors compaction of the chromatin structure, thus increasing DNA resistance to toxicants [53].

The patterns of DNA methylation are defined early during development, with two critical waves of methylation and demethylation occurring during embryogenesis [54]. Differentiated cells develop a stable and unique DNA methylation pattern that regulates tissue-specific gene transcription, and only cytosine followed by guanine can be efficiently methylated by DNA-methyltransferase [55]. Although DNA methylation is stable, it can be modified throughout life by several factors such as ageing, lifestyle, environmental exposures, and diseases. It thus represents an adaptive phenomenon linking environmental factors and the development of pathologic phenotypes such as cancers [56]. DNA methylation changes are considered to possibly play a role also in MPM development and progression, and have therefore been suggested as a potential tool for early diagnosis as well as for prognosis [57].

Several studies have been performed to evaluate alterations of DNA methylation in mesothelioma tumor samples [36, 50, 57], but very few have focused on alteration of DNA methylation in blood as circulating marker.

Fischer et al. [58] examined the methylation of nine gene specific promoters by two-stage methylation specific PCR in serum DNA of 43 patients with malignant mesothelioma (both pleural and peritoneal). Although the analysis of single gene methylation did not influence prognosis, the combined hypermethylation of the tumor-suppressors *RAR*β, *DAPK*, and *RASSF1A* was associated with shorter overall survival.

In the Arginine Deiminase and Mesothelioma (ADAM) study (a phase 2 randomized clinical trial), the role of “pegylated arginine deiminase” (ADI-PEG20), an arginine-lowering agent, was assessed in MPM patients. Indeed, arginine deprivation is synthetically lethal in cancers, such as mesothelioma, that are argininosuccinate synthetase 1 (ASS1)-negative. Among several molecular and biochemical parameters evaluated in plasma, promoter, and gene-body methylation of *ASS1* was also assessed by methylation array. The authors reported that differential *ASS1* gene-body methylation correlated with *ASS1* protein levels, and longer arginine deprivation was associated with increased progression-free survival [59].

Guarrera et al. [60] evaluated DNA methylation levels in the whole blood as potential diagnostic markers for MPM. The study was conducted on asbestos exposed subjects, 163 MPM cases, and 137 non-MPM controls. Genome-wide methylation array allowed identifying differential methylation mainly in immune system–related genes. The top hypomethylated single-CpG island was detected in *FOXK1* gene, an interactor of *BAP1*, which was found mutated in MPM tissue and constitutionally mutated in familial MPM, as described above. The analysis allowed to identify signatures of differential methylation in DNA from whole blood, between asbestos exposed MPM cases and controls.

The very few studies performed so far make it difficult to synthetically evaluate circulating DNA methylation as a potential biomarker for MPM. In addition, some biological factors must be taken into account. First, the amount of tumor-derived DNA fraction in blood is known to change according to the dimension and the state of the tumor [61]. For instance, the lung cancer-derived DNA fraction is very low in patients with relatively large tumors (e.g., 100 cm^3^) and it is generally undetectable in patients with smaller tumors, which is even more critical [62]. Second, DNA methylation in the cellular blood fraction might be only partially correlated to the methylation pattern of cancer cells, but rather represent a nonspecific marker related to the immune response toward the tumor. These factors greatly limit the use of circulating DNA methylation as diagnostic marker. Its possible role as prognostic marker needs to be further evaluated in larger populations.

## miRNAs

miRNAs are short RNA molecules of about 22 nucleotides in length. miRNAs regulate gene expression at a post-transcriptional level, by silencing protein expression through cleavage and degradation of the mRNA transcript or by inhibiting translation [63]. A single miRNA is able to bind multiple mRNA targets (more than 100), in a sequence-specific manner, and a single mRNA target can thus be regulated by different miRNAs [64]. miRNAs play crucial roles in several physiological and pathological processes such as cell growth, differentiation, proliferation and metabolism, angiogenesis, stress response, tissue remodeling, disease, and malignancy development [65]. Unique miRNA expression profiles are associated with different cancer types [66–68], and it is noteworthy that about 50% of miRNA genes in the human genome are found in genomic regions associated with cancer susceptibility [69, 70]. miRNAs are involved also in cell-to-cell communications, as they can be released by active secretion in EVs, or more rarely by energy-free passive leakage of cellular miRNAs from disrupted cells [71].

The possibility of considering circulating miRNAs as potential diagnostic and/or prognostic biomarkers for MPM has been explored for almost ten years. The main studies evaluating miRNAs are summarized in Table 1.

**Table 1:** Studies evaluating miRNA expression levels in serum or plasma.

**Study**	**miRNA**	↑/↓^*^	**MPM**	**Controls**	**Analytical method**	**Normalization strategy**	**AUC**	**AUC_95%CI**	**Sensitivity**	**Specificity**
Santarelli et al. [72]	126	↓	44	196 AES 50 AUS	TaqMan MicroRNA assay	RNU6			0.73	0.74
Tomasetti et al. [73]	126	↓	45	20 LC 56 AUS	TaqMan MicroRNA assay	RNU6	0.75 0.89	(0.62–0.89) (0.82–0.97)		
Santarelli et al. [74]	126+Met–TM+SMSRPs		45	99 AES 44 AUS	TaqMan MicroRNA assay; circulating methylated TM DNA assay, ELISA	RNU6, cel-miR-39				
Weber et al. [75]	103	↓	23	17 AES 25 AUS	miRNA Microarray, TaqMan microRNA assay	miR-125a	0.76	(0.59–0.93)	0.83	0.71
Weber et al. [76]	103a−3p+Mesothelin		43	52 AES	TaqMan MicroRNA assay	miR-125a	0.90		0.86	0.85
Cavalleri et al. [77]	103a−3p+30e−3p	↓	23	19AES	OpenArray qRT-PCR, Custom TaqMan Low Density Array	RNU48, average of miR-99a, miR638, miR-720, miR-1274a	0.94	(0.87–1.00)	0.95	0.80
Weber et al. [78]	132–3p	↓	22	44 AES	TaqMan Low Density Array Human MicroRNA Card A v2.0; TaqMan microRNA assays	miR-20b, miR-28-3p, and miR-146b-5p	0.75	(0.63–0.88)	0.86	0.61
Weber et al. [78]	132–3p+126	↓	22	44 AES	TaqMan microRNA assays	miR-20b, miR-28-3p, and miR-146b-5p			0.77	0.86
Mozzoni et al. [79]	16, 17, 126, 486	↓	32	14 ASB 15 NPD	TaqMan MicroRNA assay	miR-146 for plasma				
Matboli et al. [80]	548a−3p+20a	↑	60	20 ASB 20 AUS	RT-PCR SYBR Green PCR	RNU6B			1.00	0.87
Bononi et al. [81]	197–3p, 1281, 32–3p	↑	20	10 AES 10 AUS	miRNA Microarray; RT-PCR SYBR Green PCR	miR-3665				
Kirschner et al. [82]	625–3p	↑	30	14 AUS 10 ASB	miRNA Microarray; TaqMan microRNA assay	miR-16	0.82 0.79	(0.67–0.98) (0.66–0.93)	0.73 0.70	0.79 0.90
Kresoja-Rakic et al. [83]	625–3p+lncRNA GAS5		32		RT-PCR SYBR Green Assay	miR16-5p, beta-Actin				
Lamberti et al. [84]	101, 25, 26b, 335, 433	↑	14	10 AUS	TaqMan microfluidic cards; TaqMan microRNA assay	miR-16				
Matboli et al. [85]	2053+ lncRNARP1–86D1.3+ DRAM1+ ARSA		60	20 AES 20 AUS	RT-PCR SYBR Green Assay	ACTB, RNU6	0.94		1.00	0.85

* *Increase (↑) or decrease (↓) in miRNA expression*.

*MPM, Malignant mesothelioma; AES, Asbestos exposed subjects; AUS, Asbestos unexposed subjects; ASB, asbestosis; NPD, Noncancerous pulmonary diseases; LC, Lung cancer*.

Santarelli et al. [72] showed a reduced miR-126 expression in serum samples of MPM patients in comparison with either asbestos exposed subjects or unexposed healthy controls. The discrimination power among groups was however moderate (Sensitivity = 60%, Specificity = 74%, and Sensitivity = 73%, Specificity = 74%, respectively). A subsequent study by Tomasetti et al. [73] confirmed miR-126 downregulation in serum from both 45 MPM and 20 Non-Small-Cell Lung Carcinoma (NSCLC) patients when compared to 56 healthy controls. Furthermore, low levels of miR-126 were strongly associated with worse prognosis in MPM patients.

miR-103 was reported to be significantly down-regulated in the blood cell fraction of 23 patients with MPM, compared to 17 subjects formerly exposed to asbestos, and 25 controls from the general population. The observed differential expression levels allowed discriminating between mesothelioma patients and asbestos-exposed controls with a sensitivity of 83% and a specificity of 71%. Sensitivity and specificity for discrimination between mesothelioma patients and healthy unexposed controls were 78 and 76%, respectively [75].

Our research group was the first to investigate miRNA expression in plasmatic EVs. EVs are membrane-surrounded structures released by all cell types under both physiological and pathological conditions. EVs facilitate intercellular communication processes, as they are able to transfer biologically active molecules such as DNA, RNA, miRNAs, proteins, and lipids [86, 87]. We investigated 23 MPM patients and 19 cancer-free subjects with past asbestos exposure, and found a two miRNA (miR-103a-3p and miR-30e-3p) signature able to discriminate the two groups with high sensitivity (95.5%) and specificity (80%) [77].

The expression of miR-625-3p was significantly higher in plasma/serum of 30 MPM patients and allowed to discriminate between cases and controls (the latter consisting of 14 healthy subjects and 10 subjects with asbestosis), in both the original and in an independent series of patients. miR-625-3p was also found upregulated in tumor samples from 18 MPM patients vs. nonmalignant pleura samples, suggesting a potential connection between circulating miRNAs and the tissue of origin [82].

Weber et al. [78] found reduced miR-132-3p expression levels in the plasma of 22 MPM patients compared to 44 asbestos-exposed controls, with a sensitivity of 86% and a specificity of 61%. In order to improve the marker performance, they also measured two miRNAs (miR-126 and miR625-3p) previously reported as possible biomarkers for MPM [72, 73]. Only miR-126 was significantly differentially expressed between the two groups. The combination of miR-132-3p and miR-126 improved the diagnostic performance.

Mozzoni et al. [79] quantified miRNA-16, miRNA-17, miRNA-126, and miRNA-486 in the plasma of 32 MPM patients, 14 subjects with asbestosis, and 15 subjects with other non-neoplastic pulmonary diseases. In addition, miRNA expression was evaluated in 24 formalin-fixed, paraffin-embedded tissues (FFPE) of the MPM subjects. All investigated miRNAs were downregulated in the plasma of subjects with MPM or asbestosis compared to the healthy subjects. Furthermore, the expression of miRNA-16 in both plasma and tissue was positively related with cumulative survival.

Serum miR-548a-3p and miR-20a levels were assessed in 60 newly diagnosed MPM patients, 20 asbestos exposed subjects without MPM and 20 healthy subjects. Significant overexpression of miR-548a-3p and miR-20a was observed in MPM compared with asbestos exposed and healthy control groups, and the combined serum miRNAs showed good sensitivity and specificity [80].

Bononi et al. [81] conducted a small study on 10 MPM patients, 10 past exposed workers, and 10 healthy controls. They found upregulated miR-197-3p, miR-1281, and miR-32-3p in the sera of patients with MPM compared to those of healthy controls, upregulated miR-197-3p and miR-32-3p in MPM compared to past-exposed workers, and upregulated miR-1281 in both MPM and exposed workers compared to healthy subjects.

Lamberti et al. [84] evaluated miRNA expression in the serum of 14 MPM patients, and 10 control subjects with non-neoplastic pleural effusions. Five miRNAs (miR-101, miR-25, miR-26b, miR-335, and miR-433) were upregulated, while two (miR-191, miR-223) were downregulated. miR-29a and miR-516 were expressed exclusively in MPM patients. Based on these findings, the authors proposed two miRNA signatures characterized by different combinations of down- and up-regulated miRNAs predicting histotype and survival, but the small sample size prevents firm conclusions.

Despite the intense research activity, the translation of these results from research to clinical practice is still problematic [88]. First of all, most studies were cross-sectional and involved late stage patients, leaving open the real role of miRNAs as early diagnostic markers. Moreover, other factors hamper the reproducibility of the results, such as the limited sample sizes and the different selection criteria of controls (unexposed healthy subjects, asbestos exposed subjects with benign diseases, other cancer patients, etc.). Further inconsistencies derive from the lack of standardization in laboratory methods, techniques (types of platform, standardization procedures, and validation) and the still poor knowledge of the factors that influence miRNAs (hemolysis, age, sex, BMI, etc.). In particular, the studies previously described took advantage of different types of analytical methods and platforms (Table 1). Moreover, each study adopted different normalization strategies, thus curbing the translational potential of the obtained results (Table 1). The majority of the cited studies used a single gene as normalizer. However, single reference miRNA is not sufficient to obtain reliable data, and the combination of several normalizers may be more appropriate [89]. For instance, some studies normalized data by using the small nucleolar RNA RNU6 [72, 73], generally expressed at low and variable levels in blood and known to be altered in chronic inflammation [89–91]. Two studies used miR-16 as normalizer [82, 84], although its expression is known to be dependent on the hemolysis of samples [82]. In order to overcome the biases due to normalization, various methods have been proposed. The so-called miRNA ratio approach is based on the ratio between up- and down-regulated miRNAs within the same sample [91]. Another normalization strategy named “Global Mean Normalization” uses the average expression level of all miRNAs detected in a sample as a normalization factor [92]. For a more detailed analysis of normalization strategies in circulating miRNAs, we refer to other reviews specifically focused on the topic [92, 93].

Very recently, Weber et al. [94] tried to overcome the limitation related to the cross-sectional design by examining blood samples collected before MPM diagnosis. They analyzed three circulating miRNAs (miR-132-3p, miR-126-3p, and miR-103a-3p), previously reported as differentially expressed among MPM patients and asbestos exposed subjects, using a nested case-control approach. Seventeen mesothelioma cases were identified in a German cohort of asbestos exposed workers [MoMAr cohort [95]] for which plasma samples were available before the date of diagnosis, with a median time between sample collection and diagnosis of about 9 months. Each case was matched with two cancer-free controls by age, gender, date of blood collection, and smoking status. None of the analyzed miRNAs was differentially expressed between the two groups, and no differences in miRNA expression was detected when cases were stratified by time between sample collection and date of diagnosis. Moreover, when using a high specificity cut-off (an important characteristic for early diagnostic markers to avoid false positive results), none of the cases was detected by the examined miRNAs.

These findings limit the use of miRNAs as early diagnostic markers and leave open the debate on miRNAs as suitable markers in clinical practice. Whether the detected miRNAs are specific of MPM or rather indicative of a disease status of the subjects remains an unsolved question. Just to make some examples, alteration of circulating miR-126 expression has been associated not only with MPM but also with other neoplastic conditions [e.g., NSCLC [96] or colorectal carcinoma [97]], and with non-neoplastic conditions such as diabetes [98]; in addition, altered expression of circulating miR-103a-3p was associated also with prostate cancer [99].

Moreover, most of the miRNAs altered in MPM were downregulated. The downregulation of circulating miRNAs can be strongly influenced by the tumor growth rather than representing an initial sign of the neoplastic lesion: this might limit the utility of miRNA-based liquid biopsy in detecting the presence of small cancer sites at early stages [100]. On the other hand, focusing on miRNAs which are unexpressed or low-abundant in normal conditions but become highly expressed in cancer cells could overcome this problem, allowing that even a small tumor could generate enough of a rare miRNA to be detected in blood [101].

Another puzzling issue is the concept of “circulating miRNA” itself, as different protocols of miRNA extraction result in a different selection of miRNA origins. Cell-free miRNAs circulating in the bloodstream have been found to be either associated with EVs or rather to exist in combination with a variety of proteins (e.g., lipoproteins, Ago2 protein, or other RNA binding proteins). While EV-associated miRNAs are in majority the result of an active process, in which they are sorted and packed with a functional role (either from cancer or immune cells), protein-bound miRNAs might also be the result of an uncontrolled cellular release (e.g., after cell necrosis) from any tissue within the body. This functional difference reflects the pattern of expression observed in the two components. The large majority of the studies available to date has used whole plasma or serum as a source of miRNAs: they therefore did not allow to specifically investigate cancer tissue miRNAs, but rather a generic response to tumor development.

Recently, it has been hypothesized that cancer-derived EVs may be selectively captured, in order to extract miRNA signatures representative of the cell of origin. This new perspective may be superior to whole plasma/serum analysis as it would dramatically increase the analytical specificity of the procedure and support its application as an early diagnostic tool.

## Combination Of Circulating Molecular Biomarkers

Taking into account the limitations mentioned above, many authors [74, 83, 85, 94, 102] suggested to explore together markers of different origin to overcome the poor sensitivity and specificity of single markers. These studies combine proteins (e.g., mesothelin, fibulin, and osteopontin concentration) and different epigenetic biomarkers, including DNA methylation, expression of miRNA, and also expression of long-noncoding RNAs (lncRNAs), which are non-coding RNAs mostly involved in transcriptional regulation [103].

Santarelli et al. [74] evaluated two epigenetically regulated markers in MPM (miR-126 and methylated thrombomodulin promoter, and Met-TM) together with Soluble Mesothelin-Related Proteins (SMRPs) levels in blood serum of 45 MPM patients, 99 asbestos-exposed subjects, and 44 unexposed healthy controls. The combination of the three biomarkers only slightly improved the diagnostic performance in comparison to SMRPs alone.

Weber et al. [76] combined mesothelin and miR-103a-3p expression levels. Mesothelin concentration was evaluated in plasma, while miR-103a-3p in the cellular blood fraction. The study was conducted in 43 patients with MPM and 52 health individuals previously exposed to asbestos. The combination of mesothelin and miR-103a-3p showed a good sensitivity of 95% and a specificity of 81%.

Matboli et al. [85] measured a “MPM-specific RNA-based biomarker panel” in the serum of 60 MPM newly diagnosed patients, 20 healthy workers with past asbestos exposure, and 20 healthy subjects. The panel included the DNA damage regulated autophagy modulator 1 (*DRAM1*) and arylsulfatase A (*ARSA*), together with their epigenetic regulators: the microRNA (miR-2053) and the lncRNA RP1-86D1.3. MPM patients showed a higher expression of hsa-miRNA-2035 and lncRNA-RP1-86D1.3 and a lower expression of *ARSA* and *DRAM1* (*p* < 0.001) compared to asbestos-exposed subjects and healthy controls. The diagnostic value of the combined expression analysis showed 100% sensitivity, 85% specificity, and 94% accuracy. Moreover, the authors reported that miR-2053 expression was an independent prognostic factor for progression-free survival.

Kresoja-Rakic et al. [83] evaluated the prognostic value of miR-625-3p together with the long-noncoding RNA *GAS5* in the plasma of 36 MPM patients before and after platinum neo-adjuvant chemotherapy. The combination of increased expression of miR-625-3p and decreased expression of GAS5 was significantly associated with disease progression (*p* = 0.0393). Moreover, decreased levels of *GAS5* were associated with shorter median overall and progression-free survival compared with that of patients with increased levels of *GAS5* (*p* = 0.0308).

## Conclusion

Due to the insidious onset of MPM characterized by non-specific symptoms, that often leads to a diagnosis in advanced stages and consequent poor prognosis, a great amount of studies has focused on the search for non-invasive biological indicators that might allow to shorten the diagnostic delay and be applied in high-risk subpopulations, such as those formerly exposed to asbestos.

The ideal marker (or a combination of several markers) should have some important features such as minimal invasiveness (i.e., it should be measurable in easily obtainable biological fluids such as blood), high specificity to avoid false positives in healthy subjects, sufficient sensitivity to identify subjects with MPM, and good ability to discriminate between MPM and other diseases. As a matter of fact, if used in the clinical setting for the purposes of diagnosis and differential diagnosis (i.e., not for screening), a good marker should have an excellent discriminating power between healthy and sick people and between different pathologies when applied in addition to imaging.

Notwithstanding the plethora of existing old and new biomarkers, none of the current available markers is sufficiently reliable to be used in the surveillance of subjects exposed to asbestos or in the early diagnosis of MPM. As underlined by the Helsinki 2014 Criteria, “*At this point, no specific recommendations can be made regarding these biomarkers for screening or other purposes”* [104]. The rarity of the pathology even in cohorts of subjects exposed to asbestos and the lack of clear evidence about effective treatments that lead to improvements in survival (reduction of mortality) in cases diagnosed earlier are other factors that limit the use of the markers described so far and make it problematic. Even their possible use as diagnostic markers that can guide in the selection of subjects needing further investigation to reach an early detection of the disease remains controversial.

All the issues discussed above highlight the need for validation studies on large populations, which focus on MPM in early stages, applying shared and standardized criteria to study design, sample collection and analysis, and laboratory methods. To this end, joint research programs are needed not only at national but also at transnational level, to integrate different (clinical, laboratory, epidemiological) competences and avoid an ineffective use of resources. Furthermore, taking into account the heterogeneity of malignant mesothelioma, particularly relevant could be the study of panels of markers of different biological significance, such as “mesomiRNAs” [88, 102], miRNAs so far associated with MPM, and other protein markers along with their evaluation in repeated measures that monitor symptomatic patients over time.

## Author Contributions

LF prepared What Are Circulating Biomarkers? DNA Methylation, miRNAs, and Combination of Circulating Biomarkers paragraphs. MC prepared the Conclusion paragraph and supervised the final version of the manuscript. CM prepared the Introduction. AP supervised the preparation and revised the final version of the manuscript.

### Conflict of Interest

The authors declare that the research was conducted in the absence of any commercial or financial relationships that could be construed as a potential conflict of interest.
